# Cluster analysis of clinical, angiographic, and laboratory parameters in patients with ST-segment elevation myocardial infarction

**DOI:** 10.1186/s12944-024-02128-7

**Published:** 2024-06-04

**Authors:** Oğuzhan Birdal, Emrah İpek, Mehmet Saygı, Remziye Doğan, Levent Pay, Ibrahim Halil Tanboğa

**Affiliations:** 1 Department of Cardiology, Ataturk University, Erzurum, 25240 Turkey; 2 Department of First Aid and Emergency, Health Services Vocational School, Nisantasi University, Istanbul, 34360 Turkey; 3 Department of Cardiology, Hisar Intercontinental Hospital, Istanbul, 34764 Turkey; 4 Department of Cardiology, Ardahan State Hospital, Ardahan, 75000 Turkey; 5 Department of Cardiology and Biostatistics, Nisantasi University, Istanbul, 34360 Turkey

**Keywords:** STEMI, Short-term outcome, Machine learning, Cluster

## Abstract

**Introduction:**

ST-segment elevation myocardial infarction (STEMI) represents the most harmful clinical manifestation of coronary artery disease. Risk assessment plays a beneficial role in determining both the treatment approach and the appropriate time for discharge. Hierarchical agglomerative clustering (HAC), a machine learning algorithm, is an innovative approach employed for the categorization of patients with comparable clinical and laboratory features. The aim of the present study was to investigate the role of HAC in categorizing STEMI patients and to compare the results of these patients.

**Methods:**

A total of 3205 patients who were diagnosed with STEMI at the university hospital emergency clinic between 2015 and 2023 were included in the study. The patients were divided into 2 different phenotypic disease clusters using the HAC method, and their outcomes were compared.

**Results:**

In the present study, a total of 3205 STEMI patients were included; 2731 patients were in cluster 1, and 474 patients were in cluster 2. Mortality was observed in 147 (5.4%) patients in cluster 1 and 108 (23%) patients in cluster 2 (chi-square *P* value < 0.01). Survival analysis revealed that patients in cluster 2 had a significantly greater risk of death than patients in cluster 1 did (log-rank *P* < 0.001). After adjustment for age and sex in the Cox proportional hazards model, cluster 2 exhibited a notably greater risk of death than did cluster 1 (HR = 3.51, 95% CI = 2.71–4.54; *P* < 0.001).

**Conclusion:**

Our study showed that the HAC method may be a potential tool for predicting one-month mortality in STEMI patients.

## Introduction

Acute ST-segment elevation myocardial infarction (STEMI) is among the most damaging presentations of coronary artery disease (CAD). Despite the decreasing mortality trends after the primary percutaneous coronary intervention (PCI) era for STEMI, this clinical syndrome still has crucial adverse prognostic outcomes unless it is managed promptly and properly. Defining patient characteristics at admission and adjusting treatment strategies, including medical treatment, PCI and device therapies, have profound impacts on survival [1, 2]. Risk stratification has a favorable role in deciding both the treatment strategy and the discharge time. Throughout risk determination, several patient characteristics can be combined. Moreover, various scores can be used to estimate patient prognosis. This approach is especially crucial for determining the appropriateness of therapy. The intensity and location of care can be determined to inform patients and families more clearly about adverse outcomes and prognosis [3, 4]. Hierarchical agglomerative clustering (HAC), among the machine learning (ML) algorithms, is a relatively new method utilized in this study to categorize STEMI patients with similar clinical, laboratory and angiographic features. This approach may help to understand the outcome of patients with certain phenotypic features and intensify disease management at admission and during follow-up [5–8].

## Materials and methods

### Study population and design

A total of 3205 STEMI patients admitted to the university hospital emergency clinic between 2015 and 2023 were evaluated. The baseline clinical and laboratory parameters of the patients were recorded at admission. A detailed physical examination was also performed with data regarding current smoking status; history of CAD, previous myocardial infarction (MI), previous revascularization (either surgical or percutaneous), hypertension (HT; systolic blood pressure (SBP) > 140 mm Hg and diastolic blood pressure (DBP) > 90 mm Hg in more than one measurement or under antihypertensive therapy); diabetes mellitus (DM); family history of CAD and hyperlipidemia (HL); and noncardiac diseases, such as active or chronic infection, cancer, chronic obstructive pulmonary disease, chronic autoimmune and systemic inflammatory disease, chronic kidney or liver pathology. The current use of antiplatelet drugs, betablockers (BBs), statins and angiotensin converting enzyme inhibitors (ACEIs) was also recorded. The Killip class of each patient was recorded after thorough physical examination [9]. According to the Killip classification, classes were defined as the absence of congestive heart failure findings (class I), the presence of S3 gallop or bibasilar rales or both (class II), pulmonary edema (pulmonary rales halfway up the lung fields) (class III), and cardiogenic shock (class IV). Heart rates and systolic and diastolic blood pressure (DBP) were recorded. The 12-lead ECG signal at admission was recorded at a speed and amplitude of 25 mm/s and 10 mm/mV, respectively, by an ECG device (Nihon Kohden, Tokyo, Japan) in the supine position. Electrocardiographic and clinical diagnoses of STEMI were achieved using the current criteria of the fourth universal definition of MI. New, or presumed new, 1 mm or greater ST segment elevation at point J in two or more contiguous leads other than the V_2_ and V_3_ derivations were considered to indicate STEMI with ischemic chest pain. For the V_2_ and V_3_ leads, the criteria of 2 mm or greater elevation for males ≥ 40 years old, 2.5 mm or greater ST segment elevation for males younger than 40 years, and 1.5 mm or greater elevation for females were applied [10]. The MI patterns were defined as anterior or nonanterior and included inferior, high lateral or true posterior MI. The pain-to-door (PTD), door-to-balloon (DTB) and total ischemia time (TIT) were calculated according to recent STEMI guidelines. The PTD was defined as the time between the onset of ischemic chest pain and admission to the hospital emergency service. DTB was defined as the time between hospital admission and reperfusion that provides coronary flow distal to the occlusion. TIT is the sum of patient and system time delay to wire crossing or lytic bolus [4, 11, 12]. Transthoracic echocardiography was performed at admission to measure the left ventricular ejection fraction (LVEF) and valvular function by an expert echocardiographer in all patients in the left lateral decubitus position (GE Vivid™ 8 Ultrasound Machine; GE Healthcare, Piscataway, NJ, USA). Images of the parasternal long and short axes and apical four- and two-chamber regions were taken according to the criteria of the American Society of Echocardiography [13]. Blood samples were collected from the antecubital vein via admission to measure hemoglobin (Hgb), creatinine kinase-myocardial band (CK-MB), troponin, creatinine, albumin, total cholesterol (TC), low-density lipoprotein (LDL), high-density lipoprotein (HDL) and triglyceride (TG) levels via an autoanalyzer (ARCHITECT c16000 clinical chemistry analyzer; Abbott Laboratories, Abbott Park, IL, USA). The complete blood count, including white blood cell (WBC) and platelet (PLT) counts, was measured using an automated hematology analyzer (CELL-DYN Ruby Hematology Analyzer; Abbott Laboratories). After initial evaluation in the emergency clinic, each patient was promptly transferred to the coronary catheterization unit. Coronary angiography (CAG) was performed through either the femoral or radial artery according to the physician’s discretion. A ≥ 50% stenosis in one of the major coronary arteries was assumed to be significant. The number of diseased vessels with ≥ 50% stenosis was determined angiographically and recorded. The presence of more than one diseased artery in a patient was assumed to indicate multivessel disease (MVD). Thrombolysis in myocardial infarction (TIMI) flow measurements of the culprit artery were performed by two interventional cardiologists and defined as TIMI 0 if there was complete obstruction, TIMI 1 if the contrast agent penetrated the obstruction without distal dyeing, TIMI 2 if the dye perfused the entire artery but with slow flow, and TIMI 3 if the perfusion was normal [14]. The appropriate anticoagulant (70–100 U/kg initial IV bolus of heparin or IV dose of 0.3 mg/kg enoxaparin in patients who received prehospital subcutaneous enoxaparin injection) and antiplatelet treatments (oral loading dose of 300 mg aspirin and 600 mg clopidogrel or 180 mg ticagrelor or 60 mg prasugrel at the physician’s discretion) were started. PCI for the culprit artery and, if needed, complete revascularization were performed according to the current revascularization guidelines [15]. All patients were followed and reevaluated at the fourth week after the incident event. Any cardiovascular death after discharge in one month was considered death and was defined as one-month mortality. Patients with ST elevation in their ECGs due to early repolarization, pericarditis, left bundle branch block, or Brugada syndrome, as well as those who died before coronary angiography after admission to the hospital and those who were treated initially with thrombolytics, were excluded from the study. Patients who could not be reached for re-evaluation after four weeks were also excluded from the study. All patients and their guardians were informed about the study in case of death, and written consent was obtained. The local ethics committee approved the study. The study was conducted in accordance with the Declaration of Helsinki.

### Statistical analyses

Continuous variables are presented as medians (interquartile ranges). Categorical variables are presented as counts and percentages. Baseline characteristics were compared according to cluster using the Wilcoxon signed-rank test for continuous variables and the chi-square test for categorical variables. Two clusters were created using HAC and complete linkage methods. Gower’s formula was used for calculating dissimilarities between observations. The average silhouette method was employed to determine the optimal number of clusters. The fundamental parameters considered in the cluster analysis were age (continuous), sex, DM, HT, smoking status, HL, family history, previous MI, previous revascularization, previous use of antiplatelet drugs, BB, statins and ACEis, MI pattern, PTD, DTB, TIT, KILLIP class, SBP, DBP, heart rate, WBC, PLT, creatinine, albumin, CK-MB, troponin, TC, LDL, HDL, TG, LVEF, MVD, and TIMI flow. One-month mortality risk and cluster relationships were evaluated using Kaplan‒Meier analysis, the log-rank test, and Cox proportional hazard regression models. The associations between one-month mortality risk and patient clusters were measured using hazard ratios (HRs) and 95% confidence intervals (CIs). A *P* value less than 0.05 was considered to indicate statistical significance. All the statistical analyses were conducted using R Studio version 3.6.3 (R Project, Vienna, Austria).

## Results

**Fig. 1:**
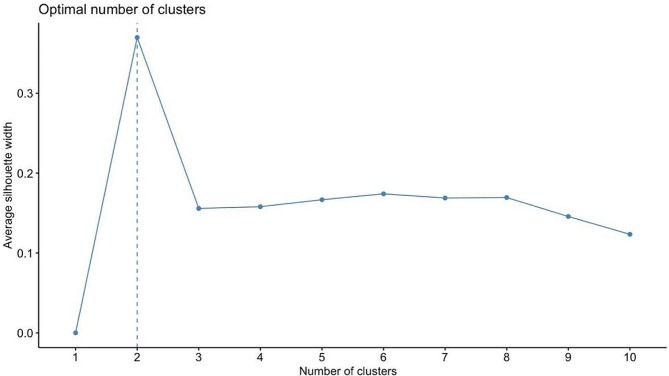
Elbow plot for determining the optimal number of clusters

**Fig. 2:**
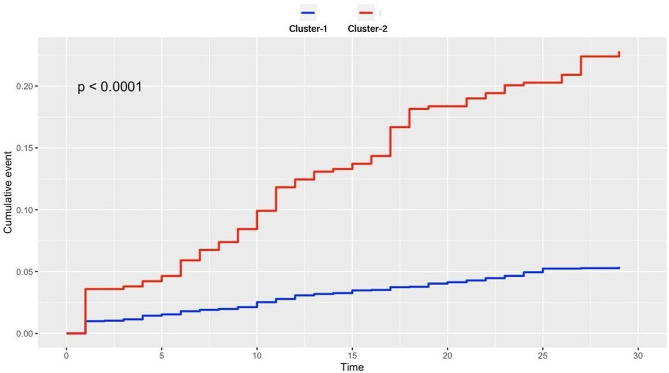
Kaplan‒Meier plot for long-term survival between clusters

A total of 3205 STEMI patients were included in our study. The basic clinical, demographic and laboratory features of the study groups and their comparisons are summarized in Table 1. The median age of these patients was 58 years. A total of 74% of the patient population was male. Fifty-six patients were in Killip class IV at presentation. A total of 1542 of the patients presented with anterior MI. After CAG, 24 patients had a TIMI flow of 0, 72 patients had a TIMI flow of 1, and 226 patients had a TIMI flow of 2. According to the average silhouette method, the optimal number of clusters was determined to be two (Fig. 1). There were 2731 patients in cluster 1 and 474 patients in cluster 2. Patients in cluster 1 were younger than those in cluster 2. There were fewer patients with HT, diabetes, and HL and fewer smokers in cluster 1 than in cluster 2 (*P* < 0.001 for all). However, the rate of previous revascularization was lower in cluster 2. In cluster 2, patients used more BBs, statins and ACEIs than did patients in cluster 1. Patients in cluster 2 had higher heart rates and SBP than did those in cluster 1. Patients in cluster 2 had higher WBC counts, PLTs and creatine, CK-MB and troponin levels; additionally, they had lower albumin and Hgb levels. Patients in cluster 2 had lower LVEFs. The number of patients with Killip class 4 and TIMI 0 flow was greater in cluster 2 than in cluster (1) Anterior MI was more common in cluster (2) At the one-month follow-up, a total of 255 patients died. Among the patients who died, 147 (5.4%) were in cluster 1, while 108 (23%) were in cluster 2 (chi-square *P* < 0.01). Survival analysis revealed that cluster 2 had a notably elevated risk of mortality compared to cluster 1 (Fig. 2) (log-rank *P* < 0.001). Cox proportional hazards regression analysis was performed to evaluate the associations between one-month mortality risk and patient clusters. Cluster 2 was associated with a greater increase in the risk of death than cluster 1 (hazard ratio (HR) = 4.65, 95% CI = 3.63–5.96; *P* < 0.001). Even after adjusting for age and sex in the Cox proportional hazard model, the statistical significance of the association between clusters and death persisted (HR = 3.51, 95% CI = 2.71–4.54; *P* < 0.001).

**Table 1:** Baseline demographic, clinical and laboratory characteristics and comparison of clusters

VARIABLES	All (*n* = 3205)	Cluster-1 (*n* = 2731)	Cluster-2 (*n* = 474)	*p* value
Sex, male n (%)	2,381 (74%)	2,113 (77%)	268 (57%)	< 0.001
Age	58 (50, 67)	57 (49, 66)	63 (54, 73)	< 0.001
DM	751 (23%)	456 (17%)	295 (62%)	< 0.001
HT	1,376 (43%)	1,054 (39%)	322 (68%)	< 0.001
Smoking	1,725 (54%)	1,603 (59%)	122 (26%)	< 0.001
HL	1,296 (40%)	1,047 (38%)	249 (53%)	< 0.001
Family history	695 (22%)	611 (22%)	84 (18%)	0.023
Previous MI	379 (12%)	327 (12%)	52 (11%)	0.532
Previous revascularization	384 (12%)	352 (13%)	32 (6.8%)	< 0.001
Antiplatelet	449 (14%)	392 (14%)	57 (12%)	0.178
BB	383 (12%)	291 (11%)	92 (19%)	< 0.001
Statin	626 (20%)	466 (17%)	160 (34%)	< 0.001
ACEIs	644 (20%)	468 (17%)	176 (37%)	< 0.001
MI pattern	1,542 (48%)	1,158 (42%)	384 (81%)	< 0.001
PTD	150 (90, 238)	145 (90, 230)	188 (125, 295)	< 0.001
DTB	30.0 (25.0, 33.0)	30.0 (25.0, 33.0)	30.0 (26.0, 33.0)	0.144
TIT	182 (123, 265)	175 (119, 257)	222 (152, 326)	< 0.001
Killip class				< 0.001
1	2,674 (83%)	2,369 (87%)	305 (64%)	
2	339 (11%)	268 (9.8%)	71 (15%)	
3	136 (4.2%)	78 (2.9%)	58 (12%)	
4	56 (1.7%)	16 (0.6%)	40 (8.4%)	
SBP	131 (118, 142)	130 (119, 140)	135 (112, 152)	0.005
DBP	78 (68, 86)	78 (68, 85)	80 (61, 90)	0.062
Heart rate	78 (69, 87)	78 (69, 85)	85 (75, 95)	< 0.001
WBC	11.9 (9.9, 14.2)	11.8 (9.8, 14.0)	13.1 (10.3, 16.0)	< 0.001
HGB	13.90 (12.55, 14.80)	13.95 (12.95, 14.80)	13.20 (11.30, 14.70)	< 0.001
PLT	260 (221, 305)	258 (220, 301)	276 (230, 318)	< 0.001
Creatinine	0.89 (0.78, 1.02)	0.88 (0.78, 1.00)	0.92 (0.80, 1.19)	< 0.001
Albumin	3.70 (3.45, 4.00)	3.70 (3.50, 4.00)	3.60 (3.20, 3.97)	< 0.001
CK-MB	176 (103, 298)	166 (99, 274)	277 (144, 412)	< 0.001
Troponin I	78 (39, 154)	73 (37, 138)	145 (66, 249)	< 0.001
TC	177 (151, 198)	177 (153, 198)	176 (143, 204)	0.187
LDL	112 (89, 135)	112 (90, 135)	111 (81, 142)	0.460
HDL	38 (31, 44)	38 (32, 44)	36 (30, 43)	0.046
TG	122 (89, 165)	123 (89, 165)	118 (84, 163)	0.108
LVEF	47 (41, 53)	48 (43, 54)	39 (34, 48)	< 0.001
MVD				< 0.001
1	1,858 (58%)	1,621 (59%)	237 (50%)	
2	957 (30%)	798 (29%)	159 (34%)	
3	390 (12%)	312 (11%)	78 (16%)	
TIMI flow				< 0.001
0	24 (0.7%)	14 (0.5%)	10 (2.1%)	
1	72 (2.2%)	50 (1.8%)	22 (4.6%)	
2	226 (7.1%)	160 (5.9%)	66 (14%)	
3	2,883 (90%)	2,507 (92%)	376 (79%)	

Abbreviations: DM: diabetes mellitus, HT: hypertension, HL: Hyperlipidemia, MI: Myocardial infarction BB: beta-blocker, ACEIs: Angiotensin converting enzyme inhibitors, PTD: Pain-to-door, DTB: Door-to-ballon, TIT: Total ischemia time, SBP: Systolic blood pressure, DBP: Diastolic blood pressure WBC: White blood cell, HGB: Hemoglobin, PLT: Platelet, CK-MB: Creatinine kinase-myocardial band, TC: Total cholesterol, LDL: Low-density lipoprotein, HDL: High-density lipoprotein, TG: triglyceride, LVEF: Left ventricular ejection fraction, MVD: Multivessel disease, TIMI: Thrombolysis in myocardial infarction

## Discussion

This study utilized an ML-based method, HAC, and identified two different phenotypical STEMI clusters with several differences in terms of laboratory and clinical findings and one-month mortality. In contemporary medicine, high-performance in silico algorithms, which have the ability to improve diagnosis and risk stratification and provide more individualized clinical management, are continuously being introduced [5]. Recently, ML algorithms have begun to be used more commonly in several clinical fields to choose the optimal therapy, especially in radiologic imaging, and it is foreseen that they will be disseminated into all medical subspecialties, including cardiology [5–8]. HAC is an ML algorithm and is categorized under unsupervised learning (UL). If we have data without specific outcomes or labels, UL is utilized [5]. HAC allows us to classify data into distinct groups. In other words, while individuals with similarities accumulate in certain clusters, individuals with dissimilarities are excluded from cluster subgroups [6]. This statistical method is included in the unsupervised analysis category without predefined groups or classes. After the gradual accumulation of subjects progresses, one specific class is formed [6]. Grouping patients into significant clusters according to their disease or clinical findings is common in medicine. This method has several advantages over other ML algorithms, which propose a general and compact data representation [7, 8, 16]. Newly learned characteristics such as group membership add more similarity information than raw patient features [17]. The lowest number of clusters determines the optimal number of groups. This increases homogeneity in the cluster and heterogeneity between the clusters, thus resulting in compatible clinical interpretation [5].

Herein, we identified two different clusters with statistically significant differences in terms of one-month mortality and survival by several analyses. This finding is not surprising when we examine the clusters in detail. Most but not all traditional risk factors [18, 19], such as male preponderance, older age, DM, HT, and HL, accumulated in cluster 2. One modifiable and one nonmodifiable risk factor, namely, current smoking status and family history of MI in a first-degree relative, seemed to accumulate in cluster 1. This can be explained by the fact that less smoker accumulation occurred in cluster 2 due to ex-smokers because we used the smoking parameter as the current smoking status. Another explanation for both risk factors may be that some cases of ischemic heart disease and STEMI occur without any definable risk factor or that only one traditional risk factor may be present [19, 20]. The increased use of BBs, ACEis and statins in cluster two paralleled the increased accumulation of HTs, DMs, and HLs in this cluster. The rate of previous revascularization was greater in cluster 1 than in cluster 2, and there was no significant difference in terms of previous MI. In the study by Bench TJ et al., 5.6% of STEMI patients had prior coronary artery bypass (CABG), 15.7% had prior PCI, and 78.7% had no history of previous coronary revascularization. In that study, patients who underwent CABG had more traditional risk factors with worse clinical outcomes than did those with previous PCI and no history of PCI [21]. In our study, the number of previous PCI patients with a relatively lower number of risk factors may be greater than that of previous CABG patients, and these patients were included in cluster (1) Increased WBC counts and adverse effects on outcomes, including infarct size and mortality, in STEMI patients have been shown in various clinical trials [22–24]. The increase in patients with higher WBC counts in cluster 2, which is the more complex and prognostically poor class, is consistent with the findings of previous studies [22, 23]. Platelets also play a crucial role in the pathogenesis of STEMI and are associated with a worse prognosis [25, 26]. A higher WBC and PLT may also signify a greater rate of inflammation in cluster (2) Because inflammation is proposed to be one of the most important factors in atherosclerosis, these findings are promising [27]. In some recent studies, a novel index of nutritional status and inflammation was introduced which is called HALP score. HALP score is calculated by the formula: hemoglobin (g/L) x albumin (g/L) x lymphocyte count (/L) / PLT count (/L).  It was reported that as this score decreases, in-hospital mortality in patients with STEMI and all-cause mortality in patients with CAD increase [28, 29]. The relatively lower albumin and hemoglobin levels and higher platelet counts in cluster 2 may therefore be related to the greater number of patients at greater risk. The higher creatinine levels may be a result of the increased number of diabetic individuals in cluster 2. Lipids play crucial roles in the pathophysiology of plaque formation and rupture in patients with STEMI [18, 20]. The serum levels of TC, LDL and TG were not significantly different between the clusters. This may be because of the significantly greater number of patients with statin use in cluster 2. A significantly lower HDL-C level may also reflect the greater proportion of patients with a relatively higher risk in cluster 2.

Current guidelines recommend certain time frames for proper management of STEMI patients [11, 30]. The minimum delay after the onset of chest pain to lytic bolus or wire crossing in the culprit artery is crucial for preventing life-threatening STEMI complications such as ventricular arrhythmias and in-hospital and long-term mortality [30–34]. This is achieved by both community awareness of coronary ischemic symptoms and an efficient medical system that takes care of STEMI patients. The PTD was reported to be related to socioeconomic factors and sex [31]. According to a previous meta-analysis, female patients with lower rates of primary PCI have a significantly longer time lag since initial medical contact and greater DTB [35]. PTD time was significantly lower in cluster one, in which the number of males was significantly greater (77%), than in cluster 2. This can also be explained by the uneven number of females in our study. In cluster 2, we observed increased PTD, which contributed to the increased complexity of this class in our study. The similar DTB times in both clusters may be due to the single-center nature of the study. Additionally, the established standard 7/24 continuous PCI capability and advanced triage system of our institution may have played a role in our relatively lower DTB times in both clusters, which had a median of 30 min compared with the recommended ≤ 90 min. The absence of chest pain and not using an ambulance were reported to be related to failure to reach the DTB target [32].

The increase in patients with higher SBP and heart rate in cluster 2 was a remarkable finding. The relatively higher heart rates and SBP can be explained by the presence of more anterior MIs in cluster 2. A previous meta-analysis showed that while the sympathetic nervous system (NS) dominates over the parasympathetic NS in anterior MIs, the vagal system predominates in inferior MIs [36]. Pre-PCI angiographic TIMI flow in the culprit artery is an important parameter that is related to infarct size and microvascular obstruction [37]. It was shown to be strongly related to severity and major adverse events and was reported to be an independent predictor of increased survival at one year [14, 37–39]. In STEMI patients, initial better TIMI flows (≥ 2) were reported to be more frequent in recent years, reaching 40% [40]. In our study, the relatively greater TIMI 2 and 3 flows in general may be attributable to appropriate prehospital management, including timely administration of antiplatelet therapy and heparin. Nevertheless, patients with TIMI 2 and 3 flowsignificantly accumulated in cluster 1. A greater number of patients with TIMI 0 or 1 flow in cluster 2 may contribute to greater infarct size and greater LV remodeling and dysfunction [37, 41, 42, 43], which increases the risk in cluster 2 compared with cluster 1. A significantly greater number of patients with anterior MI and higher CK-MB and troponin levels in cluster 2 support the associations discussed above.

The Killip class was independently associated with mortality, in-hospital cardiac arrest and acute renal failure in MI patients. The risk of mortality seems to persist at one month and five years [43]. The mortality risk according to the Killip classification was reported to be maintained even after adjustment for physical and clinical variables, including SBP, resting heart rate, age and several comorbid situations, including DM and previous revascularization [9, 43].

Multivessel coronary disease is detected in nearly 50% of patients with acute coronary syndrome. It was reported to be significantly associated with poor outcomes and increased mortality [44]. With a similar pattern of accumulation, the number of patients with 2- and 3-vessel disease was greater in cluster 2.

One of the most interesting findings in our study was that clinical presentation at admission has a substantial impact on prognosis. Owing to the greater number of patients with a higher KILLIP class and lower LVEF, an increased number of anterior MIs and increased MVD in cluster two resulted in a greater mortality rate with decreased survival within one month. Additionally, even after adjusting for age and sex, the association between clusters and death persisted. These findings are consistent with previous research [34, 35, 42]. In our study, we identified two phenotypically distinct STEMI clusters. This approach may be particularly helpful for stratifying patients and adjusting appropriate medical and interventional treatments, including complete revascularization during primary PCI and earlier/late discharge from the coronary intensive care unit and hospital. Patients in cluster 2 may need more frequent follow-up than patients in cluster 1.

### Study strengths and limitations

One of the strengths of this study is that it studies a novel ML algorithm, HAC, for the categorization of individuals at increased risk of developing STEMI. In terms of AI use in medicine, this study may encourage the development of models that utilize AI for more accurate risk determination in certain medical situations. There are several limitations in our study. First, due to the retrospective nature of our study, there is a potential for bias, which is common in retrospective research. Second, despite being a high-volume clinic with a sufficient number of patients, differences in operator experience may have an impact on patient outcomes.

## Conclusion

This study used a relatively new ML-based method, HAC, and revealed two different phenotypical disease clusters. There are several differences between the two STEMI clusters in terms of laboratory and clinical findings and one-month mortality. The admission and in-hospital parameters were worse in cluster 2, while there was a relatively benign accumulation in cluster 1. Our study is among the first analyses of clustering approaches in a previously well-studied patient cohort. Despite significant progress in medical therapy and timely PCI, it is noteworthy to analyze STEMI patients from a different point of view despite continuing to have high morbidity and mortality.

## Data Availability

No datasets were generated or analysed during the current study.
